# The fallacy of global comparisons based on *per capita* measures

**DOI:** 10.1098/rsos.230832

**Published:** 2024-03-20

**Authors:** Lukáš Kratochvíl, Jan Havlíček

**Affiliations:** ^1^ Department of Ecology, Faculty of Science, Charles University, Viničná 7, Prague 128 00, Czech Republic; ^2^ Department of Zoology, Faculty of Science, Charles University, Viničná 7, Prague 128 00, Czech Republic

**Keywords:** ratios, global comparisons, gross domestic product, *per capita* measures

## Abstract

Media, social scientists and public health researchers often present comparisons across countries, and policy makers use such comparisons to take evidence-based action. For a meaningful comparison among countries, one often needs to normalize the measure for differences in population size. To address this issue, the first choice is usually to calculate *per capita* ratios. Such ratios, however, normalize the measure for differences in population size directly only under the highly restrictive assumption of a proportional increase of the measure with population size. Violation of this assumption frequently leads to misleading conclusions. We compare *per capita* ratios with an approach based on regression, a widely used statistical procedure that eliminates many of the problems with ratios and allows for straightforward data interpretation. It turns out that the *per capita* measures in three global datasets (gross domestic product, COVID-19-related mortality and CO_2_ production) systematically overestimate values in countries with small populations, while countries with large populations tend to have misleadingly low *per capita* ratios owing to the large denominators. Unfortunately, despite their biases, comparisons based on *per capita* ratios are still ubiquitous, and they are used for influential recommendations by various global institutions. Their continued use can cause significant damage when employed as evidence for policy actions and should therefore be replaced by a more scientifically substantiated and informative method, such as a regression-based approach.

## 1. Wide use of *per capita* measures and general problems with ratios

The comparisons of countries in a relative measure appear to be ubiquitous. Local media are regularly informing their readers that our home country, the Czech Republic, is, after normalization for population size, a champion in beer consumption [1], that it was one of the worst in COVID-19-related mortality during the pandemic [2], or that Czechia jumped over Spain but is beyond Slovenia in this or that economy-related measure [3]. Similar examples can be taken from the major media globally, as can be illustrated by selected phrases after entering ‘*per capita*’ in the search engine, such as ‘Wyoming has more guns *per capita* than any other state’ [4], ‘(did you know) the US has about the same # of MD specialists *per capita* as the UK? And fewer than Slovenia, Latvia, Estonia, Italy, & Hungary?’ [5] or ‘given that the U.S. is the world’s largest producer of oil and gas, the most responsible for global cumulative emissions, and one of the globe’s biggest *per capita* emitters today, it must get its renewable and reparative ducks in a row. …’ [6]. Global comparisons among countries are not restricted to popular media but they are common in research literature as well, particularly among social scientists and public health researchers [7,8]. Policy makers then often use such comparisons to take evidence-based action [9,10].

The main reason why relative values such as *per capita* are so widespread is that absolute values are mostly not of much use for comparison among countries because they vary in their population size. For a meaningful comparison, one needs to control for differences in population size (i.e. normalize). To address this issue, the first choice is usually to calculate the ratio between the measure of interest and the population size and then compare the values of *per capita* measures among countries. This intuitively appealing and seemingly straightforward procedure might, however, lead to misleading conclusions [10].

**Figure 1.**
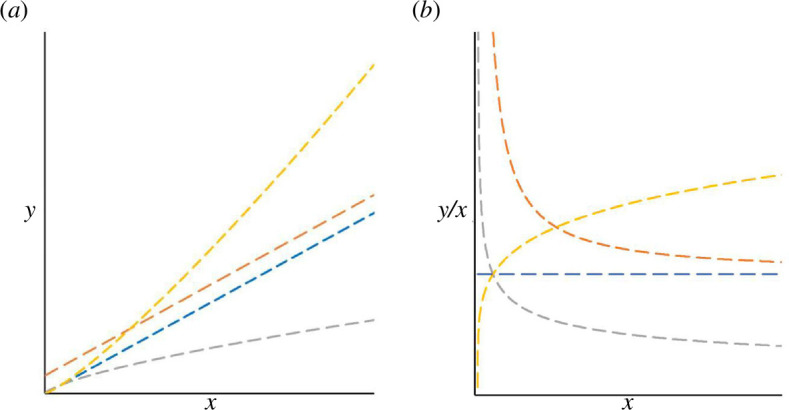
Exemplary increasing functions describing relationships between variables *x* (e.g. population size) and *y* (e.g. a country measure) (*a*), and the dependence of the corresponding *y*/*x* ratios on the argument *x* (*b*). The only function that gives a constant when divided by its argument is the linear function going through the origin (blue lines). Ratios thus properly standardize *y* for variability in *x* only when *y* increases in direct proportion to *x*. Note that in the other depicted cases, such as when the linear relationship between *y* and *x* does not go through the origin (orange) or an increase of *y* with *x* according to a power function (with power >1 in yellow and between 0 and 1 in grey), the ratios have an infinity (or minus infinity) limit as *x* tends to zero. As a consequence, even a small shift in *x* in small values produces a large change in the ratio *y*/*x*.

Some of the problems with ratios were already identified at the dawn of statistics as a science [11,12]. The major issue lies in the fact that the sole function yielding a constant when divided by its argument and thus removing the effect of a denominator is a linear function that goes through the origin of the coordinate system (figure 1). *Per capita* ratios can be directly used for comparisons across countries only when the measure of interest increases proportionally with population size. In all other cases, ratios ought to be interpreted with extreme caution owing to their various non-intuitive statistical properties, including: (i) a frequently strong departure from normal distribution, (ii) asymmetry with respect to changes in the numerator and denominator, and (iii) scaling with the denominator (figure 1).

Serious problems with ratios in comparative studies have been recognized in various branches of life sciences, including morphometry [12], evolutionary genetics [13] and ecological stoichiometry [14]. In some of them, the use of ratios has been largely abandoned and replaced by more appropriate procedures [12,15]. Nevertheless, global comparisons of various aspects of economic performance, public health or environmental impact are still frequently based on *per capita* indicators [16,17]. Measures standardized in this way suffer from the above-mentioned issues of ratios and frequently lead to worryingly misguided outcomes. Given the global impact of these measures, we believe there is an urgent need for alternatives.

## 2. Regression-based approach as an alternative to *per capita* measures

For normalization, we propose a widely used approach based on linear regression, which also provides simple and appealing measures but does not suffer from many of the problems that ratios have. We compare these two approaches by applying them to three exemplar global comparisons: gross domestic product (GDP; data from https://datacatalog.worldbank.org), COVID-19-related mortality (data from https://www.worldometers.info/coronavirus/#countries) and CO_2_ production (data from https://www.worldometers.info/co2-emissions/). Specialists in the relevant fields extensively discuss the pros and cons of these measures. For instance, it is largely recognized that GDP need not be the best proxy for a country’s economic performance and progress [17,18]. Similarly, data on COVID-19-related mortality may suffer from systematic inaccuracies related to differences in data gathering and publication among countries [19,20]. However, these variables are still widely used and affect political decisions in economy, health systems, environment conservation as well as other areas [21,22]. At present, we shall focus on the problems of *per capita* comparisons and leave aside other questions concerning the suitability of particular measures (for a discussion, see [18,23]).

**Figure 2.**
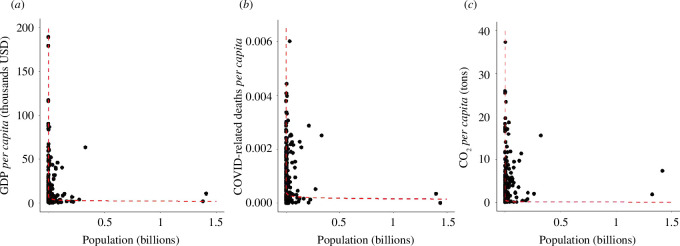
Plot of *per capita* measures against population size across countries. The *per capita* ratios in all three measures*:* (*a*) GDP, (*b*) COVID-19-related deaths, and (*c*) CO_2_ emissions exhibit enormous variability among countries with small populations. This variability then decreases with increasing country population size. This is owing to the infinity limits of the underlying functions as population size approaches zero. The red lines represent predictions for ratios derived from the back-transformation of the linear relationship between logarithmically transformed values of each measure and population size depicted in figure 3*b*.

**Figure 3.**
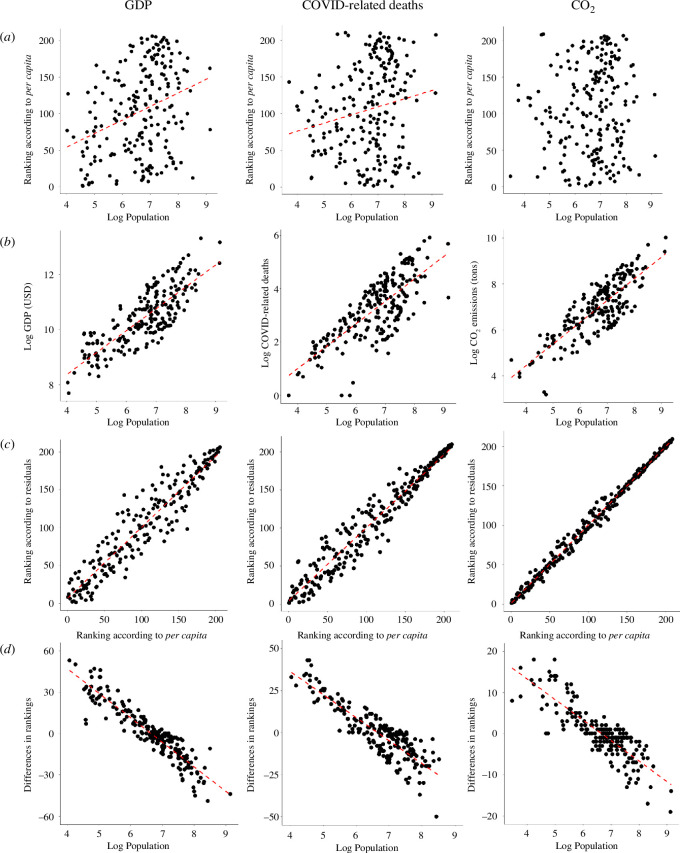
Scatterplots comparing country rankings based on *per capita* ratios and residuals. (*a*) Rankings according to *per capita* ratios significantly increase with population size in two out of three measures, demonstrating that the ratios do not really control for population size. (*b*) The increase of measures with population size. (*c*) Rankings based on ratios and residuals are correlated. (*d*) Differences between rankings based on *per capita* ratios and residuals highly correlate with population size, which indicates that *per capita* ratios systematically overestimate values in countries with small populations and underestimate them in countries with large populations.

The *per capita* expressions in all three measures (GDP, COVID-19 mortality and CO_2_ production) show a pattern typical of many ratios. Firstly, the ratios substantially deviate from normal distribution (electronic supplementary material, figure S5). The mean *per capita* value, while often used to describe the central tendency, is therefore not an informative representative of the dataset. Secondly, the ratios exhibit enormous variability among countries with small populations; this variability then sharply decreases with countries’ increasing population size (figure 2). Small changes in population size thus have disproportionately larger effects in countries with small populations. Thirdly, *per capita* GDP and *per capita* COVID-19-related mortality show a negative correlation with population size (Spearman’s *ρ* = −0.32, *p* < 0.00001 and *ρ* = −0.20, *p* < 0.004, respectively). The data on GDP and COVID-19-related mortality thus do not increase proportionally with population size, and the *per capita* ratios do not adequately standardize for differences in population size. This is further reflected in a positive correlation between country ranking according to *per capita* GDP and *per capita* COVID-19 death rates on the one hand and population size on the other hand (Spearman’s *ρ* = 0.32, *p* = 0.000002, and *ρ* = 0.20, *p* = 0.004, respectively, figure 3*a*). By contrast, the correlation between *per capita* CO_2_ emissions and population size is not statistically significant (*ρ* = −0.11, *p* = 0.10). Consequently, the correlation between a ranking based on *per capita* CO_2_ emissions and log-transformed population size is not statistically significant either (*ρ* = 0.09, *p* = 0.21). Nevertheless, the shifts in *per capita* ratios in CO_2_ emissions demonstrate a strong asymmetry with shifts in population size (electronic supplementary material, figure S5), suggesting that the *per capita* ratio is not an optimal index for this measure either.

The assumption of proportionality of a measure to the population size is rarely tested and even less frequently met. Researchers who use the *per capita* ratio to normalize a measure of population size should be aware that a violation of the proportionality assumption leads to severe biases when *per capita* ratios are used for direct comparisons among countries. A correct approach to global comparisons should not be based on unrealistic and often violated assumptions: it should consider the general trend of the data. We cannot stress enough that before using any analytical technique, one should carefully check the trends in a given dataset including its distribution, central tendency and effect of outliers. The properties of data one ought to consider are carefully well known and include nonlinearity, homoscedasticity and leverage [11].

In all three examples analysed here, the linear function of log_10_-transformed measure to log_10_-transformed population size describes the general trend well. These functions explain 65%, 59% and 68% of the total variance, demonstrating that total GDP, COVID-related mortality and CO_2_ production, respectively, scale with population size and that a standardization for population size is necessary for comparisons. The addition of a quadratic term to each of these relationships did not significantly improve the model’s explanatory power (for details, see the electronic supplementary material). We thus further neglect a slight nonlinearity in the log–log relationships.

Importantly, these linear approximations on the log–log scale explain the behaviour of the *per capita* ratios very well. To illustrate it, we back-transformed the linear functions of logarithmically transformed values and depicted the ratios of values predicted by the back-transformed power functions for a given population size (figure 2). The predicted *per capita* measures approach infinity as the population size approaches zero, which is responsible for the strong asymmetry in *per capita* ratios. Most notably, owing to the steepness of the functions as they approach zero, a small shift in the population size or the measure of interest in countries with a small population leads to an enormous shift in the *per capita* ratio. By contrast, even a large change in population size or the measure of interest in countries with large populations has little effect on the *per capita* ratios, and their ratios are, in general, relatively low. Nevertheless, regardless of the precise underlying function, in all cases, the *per capita* ratios are clearly inappropriate for direct standardization for differences in population size among countries.

## 3. Systematic differences in country rankings based on *per capita* measures and residuals

**Figure 4.**
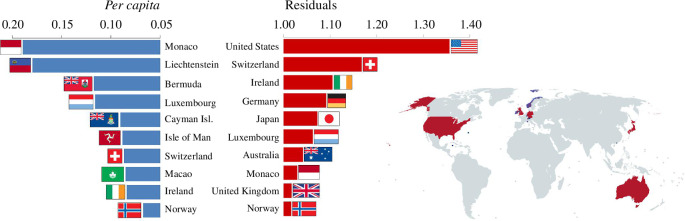
Top 10 countries in GDP controlled for population size highly differ in rankings according to *per capita* measures and residuals. Note that only countries with a small population are represented among the top 10 according to the *per capita* ratios (given as USD *per capita*), which reflects an overestimation of their measures by this procedure.

Instead of the highly problematic *per capita* ratios for comparison among countries, one may employ residuals from the log–log linear regression. These residuals have a straightforward interpretation: their values +1.0 and −1.0 correspond to 10 times higher and 10 times lower values of a measure in comparison to the value expected for a country of a particular population size (residual 0). The similarities and differences between the *per capita* approach and the regression approach can be exemplified by comparing the rankings of individual countries on a particular measure. Rankings based on the two approaches were in all three measures highly correlated, which reflects the enormous differences among countries (figure 3*c*), but we also found some important differences between the rankings. None of the rankings based on the linear regression approach correlated with the population size, which is exactly what one would expect from a procedure standardizing for differences in population size. The direction of shifts in the rankings in all three measures is highly correlated with country population sizes (figure 3*d*), which is what one would expect from the relationship between *per capita* ratio and population size (figure 2). In particular, it can be expected that some small countries will have misleadingly large ratios because of the small denominator (population size), while large countries will have misleadingly low ratios owing to large denominators substantially decreasing their metrics. We recorded the largest change in the GDP ranking relative to population size in Nauru, which moved 66 positions down, while India moved 64 positions up in the ranking based on the regression approach as compared to the *per capita* measures (electronic supplementary material, table S1). In fact, all 29 countries with a population above 50 million moved up (on average, 29 positions, which is considerable). The effect of large denominators on the *per capita* ratio can be exemplified in China and India, the two most populous countries in the world. According to the GDP *per capita* measure, both are in the broad middle of the ranking: China ranks 78th and India is even 162nd among 206 countries. However, when we compare countries without the bias introduced by the behaviour of ratios, India (with residual −0.04) reaches 91% of GDP expected for a country of its population size, and China (residual 0.70) has over five times higher GDP than predicted for an average country of its population size. In ranking according to residuals, India is around the middle of the ranking (98th place), while China is among the 17% of highest-scoring countries (34th place). Also, we can notice that only small-population countries are represented in the top 10 of the GDP ranking according to *per capita* ratios, while in ranking according to residuals, the top 10 countries are much more balanced with respect to their population size (figure 4).

Similarly, as for the GDP, three countries of small population size (Gibraltar, San Marino and Liechtenstein) versus two countries with a large population (Indonesia and India) showed the largest change in COVID-related mortality rankings, with the small countries moving up (in terms of higher mortality) and the large ones down (electronic supplementary material, table S2). Two countries with a large population (the USA and Brazil), where the situation with COVID-related mortality was apparently dramatic [24,25], took in the regression-based ranking the second and third positions (in highest mortality) right after Peru, where COVID-related mortality was so disastrous [26] that it headed the rankings based on both approaches. Again, the much lower positions of the USA and Brazil in the *per capita* ranking should be attributed to their large population size, that is, the large denominator in the calculation of the *per capita* ratio.

In the CO_2_ emission ranking, the situation was analogous, but the changes in the ranking were smaller. Still, the most notable movements in the rankings in the opposite directions were between Palau and Seychelles, which moved down, versus India and Brazil, which moved up (electronic supplementary material, table S3). Countries with a population of over 50 million inhabitants moved on average seven positions up, meaning their production of CO_2_ emissions standardized for population size is in a global comparison comparatively much higher than one would assume based on the *per capita* ranking.

## 4. Why are *per capita* ratios still so prevalent?

We are not the first to draw attention to the biases introduced by the use of ratios and the need for alternatives. For instance, Ranta *et al*. [27] claimed that a regression-based approach amounts to reinventing the wheel in their instructive demonstration of problems with ratios in comparisons of sexual size dimorphism among animals. Still, the use of *per capita* measures, an exemplary case of ratios, remains ubiquitous. For instance, the World Bank classifies the world’s economies into four income groups—low, lower-middle, upper-middle and high-income countries—based on *per capita* gross national income. Therefore, we feel compelled to stress that continued use of *per capita* measures is not only of limited informative value but can also result in misleading conclusions and cause extensive damage when employed as evidence for policy actions. The alternative offered here, the regression-based approach, is computationally simple and can be flexibly adjusted for general trends in data.

It is difficult for us to understand why the *per capita* approach is still so widely used in some fields, although the problems with ratios have been so widely discussed for decades [27–30]. We fully identify with the Jasienski & Bazzaz [30] statement: ‘Researchers love ratios. Statisticians loathe them. The simplicity of ratios, when one variable is divided by another, makes them appealing mental devices, yielding conclusions that are easy to interpret, even if erroneous, and warnings about their misuses are frequently left unheeded’. Based on our experiences, we think that there are three major reasons why ratios are still used for normalization/standardization:

- ratios are indeed very simple to calculate and are thus intuitively appealing, which gives the impression that they are simple to interpret, which is not at all true;
- a tradition in a given field that is difficult to abandon, as researchers tend to use a technique they learned during their training. This may include the use of the second- to fourth-digit ratio in biological anthropology (for recent meta-analysis, see [31]), which is still very common, although it was previously criticized [12,32]; and
- a lack of proper mathematical knowledge about functions. The problems with ratios are often not included in practical statistical/mathematical courses. For a similar issue on the interpretation of log-scale figures, see [33]. Probably, many researchers do not understand well the mathematical consequences of using ratios, and they are still not aware of their problems.

It is an interesting question why ratios were largely abandoned in some research fields, such as comparative zoology (it might be an impact of the influential paper by Atchley *et al*. [28] and subsequent discussions), while the debate is still ongoing in other fields including comparative anatomy [34], biological anthropology [12,32], evolutionary genetics [13], and maybe the debate has not even started in other fields. We call for a more interdisciplinary exchange of knowledge on analytical techniques.

We think that the major difference between researchers who use ratios and those using regression-based approaches for standardization is that the former are strictly stuck on the assumption that the relationship between a numerator and a denominator is simply a straight line going through the origin (and they are unfortunately mostly not aware that they assume it and that this strong assumption should be statistically tested before the ratio is accepted as a proper standardization procedure). On the other hand, the latter are aware that there might be an underlying mechanism driving the general pattern, but they are often not sure what mechanism drives such a pattern. One may consider this fact a major disadvantage of the regression approach. However, for a meaningful comparison of normalized or standardized values, they know that they should filter the major trend statistically to get ‘size-free’ (normalized) values.

A further disadvantage of the regression-based approach is its seeming complexity. This issue can be easily overcome by education, though regression is part of almost every basic statistical textbook [11,35]. Researchers and the general public alike need to learn an interpretation of its results, which in the case of residuals from a linear regression on log–log transformed data is not that difficult. The residuals in this case are dimensionless normalized measures expressing departures from a predicted value for a country with a given population size in a multiplicative way. The interpretation of comparisons based on regression-based techniques is thus straightforward, and the results can be thus easily communicated to the general public.

## 5. Conclusions

We believe that policy makers, practitioners and influential organizations, such as the World Health Organization [21], World Bank [22], International Monetary Fund [36], Eurostat [37] and the United Nations [38], as well as various widely used information sources such as Wikipedia [39], could adopt the regression-based techniques easily and abandon the *per capita* approach, which appears to standardize well for a population size but in fact provides in many cases distorted insights. We emphasize that these organizations not only publish the statistics in the *per capita* forms but also make a direct comparison across countries based on them with often crucial consequences. The web page of Eurostat is a striking example, even more so with its official public Tweets like ‘In 2022, Luxembourg recorded the highest level of GDP *per capita* in the EU followed by Ireland, Denmark, the Netherlands, Austria and Belgium’ [40]. We believe that the readers of this paper will become reserved for such proclamations and should be alerted when they see a very small country dominating a ranking in a GDP-related or other *per capita* measure.

## Data Availability

All data used in this work are available in public databases, which are cited in the text body [41–43]. Electronic supplementary material is available online [44].
